# Curcumin Conjugated Gold Nanoclusters as Perspective Therapeutics for Diabetic Cardiomyopathy

**DOI:** 10.3389/fchem.2021.763892

**Published:** 2021-10-26

**Authors:** Dong-zhuo Wei, Dan Li, Dan-meng Zheng, Zhen-ni An, Xue-jiao Xing, Ding-wen Jiang, Xi-fan Mei, Chang Liu

**Affiliations:** ^1^ Clinical Discipline of Chinese and Western Integrative Medicine, Liaoning University of Traditional Chinese Medicine, Shenyang, China; ^2^ Public Basic Academy, Jinzhou Medical University, Jinzhou, China; ^3^ Department of Endocrinology, The First Affiliated Hospital of Jinzhou Medical University, Jinzhou, China; ^4^ Department of Orthopedics, The First Affiliated Hospital of Jinzhou Medical University, Jinzhou, China

**Keywords:** curcumin, AuNCs, lipid metabolism, H9c2, apoptosis

## Abstract

Accumulation of lipids in the myocardium contributes to the development of cardiac dysfunctions and various chronic diseases, such as diabetic cardiomyopathy (DCM). Curcumin (Cur) can relieve lipid accumulation problems, but its efficiency is limited by poor water solubility and biocompatibility. Herein, gold nanoclusters (AuNCs) were used to improve the efficiency of Cur, and the conjugates Curcumin-AuNCs (AuCur) were developed. In the treatment of high-fat-induced myocardial cell damage, we found that AuCur could effectively reduce intracellular lipid accumulation, the increase of reactive oxygen species (ROS), the increase of mitochondrial division, and the increase of apoptosis compared with Cur. AuCur decreased the expression of the peroxisome proliferator-activated receptors-α subtype (PPARα), and the therapeutic effect of AuCur was canceled when the expression of PPARα was enhanced. For the above reasons, AuCur treated the toxic effect of high lipid on cardiomyocytes by regulating PPARα, providing a new idea and method for the treatment of DCM.

## Introduction

DCM is one of the most serious complications of diabetes mellitus (Hu et al., 2017). Lipid accumulation is one of the most important reasons causing DCM and cardiomyocytes (Goldberg et al., 2012), which increased long-chain FA transporter CD36 and PPARα (Finck et al., 2002). This suggests that inhibition of over-expressed PPARα can prevent abnormal accumulation of lipids (Wu et al., 2018). Lipid accumulation is also accompanied by an increase in reactive oxygen species in cardiomyocytes (Son et al., 2010; Li et al., 2020a). The imbalance between the production and clearance of reactive oxygen species can cause mitochondrial dysfunction and apoptosis, and other cardiac complications (Britto et al., 2018; Li et al., 2020a; Fan et al., 2020).

Cur is a natural plant extract, which is well known for its biosafety and versatility to treat a series of diseases (Govindaraju et al., 2018). The regulatory effect of Cur on lipid metabolism has attracted people’s attention (Aggarwal et al., 2013; Qi et al., 2017). However, because of the strong hydrophobic nature of this drug, which affects its biocompatibility, the uptake efficacy of curcumin is insufficient, which limited the therapy of cardiomyopathy (Zhang et al., 2019; Czyzynska-Cichon et al., 2021). Once Cur can be more efficiently used, more promising effects can be expected.

Some nanomaterials have good biocompatibility including metal oxides (ZnO and MgO) which are promisingto improve the bioavailability of Cur. However, it is challenging to obtain fluid compatible ZnO with long-term use. The sizes of ZnO and MgO are normally larger than 20 nm, which brings concerns to pass the organ barriers. Additionally, the large nanoparticles tend to accumulate to the liver, kidney, and heart organs of rats (Metzler et al., 2013; Li et al., 2020b; Matus and Häkkinen, 2021). On the other hand, Gold nanoclusters (AuNCs) with ultra-small sizes (<3 nm) show excellent fluid solubility and can be used as effective injective drugs (Li et al., 2021). They can pass various barriers in the body (Xiao et al., 2016; Mangalampalli et al., 2017; El-Sayed and Schneider, 2020; Verma et al., 2021), and have a renal clearance rate larger than 75% (Du et al., 2017). They have been used as carriers to deliver some less biocompatible drugs causing insignificant toxicity. However, it is unknown whether the herbal medicines can be safely circulated *in vivo* for the treatment of DCM. Herein, the highly stable Bovine Serum Albumin (BSA) stabilized AuNCs (BSA-AuNCs) were used to conjugate with the herbal medicine, i.e., Cur. AuCur were proved to have great potential for treating DCM for the first time. This opens an avenue to use AuNCs to improve the treatment of cardiomyopathies.

## Article Types

Original Research Articles. The length of the manuscript is 3,650 words.

## Materials and Methods

### Synthesis and Conjugation

BSA stabilized AuNCs were synthesized according to previous reports with slight modifications. Briefly, HAuCl_4_ (1 ml, 50 mM, 37°C) and 0.25 g of BSA were mixed in a 10 ml water solution. After the mixture was dissolved as a clear solution, 1 M of NaOH solution was added and the mixture was transferred to a water bath and incubated at 37°C for 12 h. The solution was filtered by a 0.22 µm filter and dialyzed against (10,000 molecular weight cut-off dialysis membrane) with three times distilled water. The solution was filtered. Then, 1 mg of Cur was dissolved in the as obtained BSA-AuNCs (1 ml). For comparison, another 1 mg of Cur was dissolved in a water solution.

### Cell Culture

H9c2 cardiomyocytes derived from rat myocardium were cultured in Dulbecco’s modified Eagle’s medium (DMEM), all of them are containing 10% fetal bovine serum and 1% penicillin-streptomycin. The culture incubator was placed in 5% CO_2_ at 37°C. We used 0.25% trypsin-EDTA (Gibco, Invitrogen, United States) to pass the cells after the cell density reached 80%. The frequency of medium change was 3 days.

### Cell Viability Assay

The MTT [3-(4, 5-dimethylthiazol-2-yl)-2, 5-diphenyltetrazolium bromide] assay was used to estimate the proliferation of H9c2 cells. Briefly, the cells were plated in 96-well plates (5 repeat wells) at a density of 1 × 104 cells/well for 24 h, the cells were serum-starved overnight in a medium containing 0.5% fetal bovine serum. Then, the different concentrations of Cur and AuCur were added for 1 day, respectively. Afterward, 20 μl of MTT solution [5 mg/ml in phosphate buffer solution (PBS)] was added into each well for 4 h. Subsequently, the supernatant was discarded, and 150 μl Dimethyl sulfoxide (DMSO) was added to each well. Shake on a shaker for 15 min. Finally, quantitative detection was performed on a microplate reader at the wavelength of 490 nm.

### Lipid Staining of Cells

*In situ* cells were stained with oil red O staining solution (G1262, Solarbio Biotechnology, Beijing, China) to observe the accumulation of lipid droplets in cells. Briefly, we removed the cell culture, then washed it with PBS 2 times, and added Oro Fixative 20–30 min fixation solution. The fixed solution was removed and washed with distilled water two times. The cells were soaked in 60% isopropanol for 5 min. Then, isopropyl alcohol was removed and a newly prepared Oro Stain was added to soak for 10–20 min. The cells were washed with water 2–5 times until there was no excess dye. Mayer hematoxylin staining solution was added and the nuclei were restained for 1–2 min. After washing the cells again 2–5 times, ORO Buffer was added for 1 min. Finally, the cells were covered in distilled water and examined under a microscope.

### Measurement of Intracellular ROS

The intracellular ROS was analyzed with ROS assay kit (S0033, Beyotime, Shanghai, China) respectively according to the manufacturer’s instructions. Briefly, We washed the cells with PBS. Then, ROS capturing reagent DCFH-DA (2,7-Dichlorodi -hydrofluorescein diacetate) was added and incubated at 37°C for 30 min without light. Finally, fluorescence confocal microscopy (Leica TSC SP5 confocal unit) was used for observation.

### Immunofluorescence Staining

In each group, H9c2 cells were washed 3 times by PBS after incubating for a certain time. Then, these cells were fixed by 4% Paraformaldehyde (PFA) for 30 min. Subsequently, the cells were washed by PBS 3 times and then blocked by 5% goat serum for 2 h. After that, these cells were incubated in a culture medium with primary PPARα (1:200, AF5301, Affinity, Biosciences) antibodies, Drp1 (1:50,8570S, Cell Signaling Technology) antibodies, and anti-β-Tubulin antibodies for staying overnight at 4°C. Then, the cells were rinsed with PBS 3 times. Subsequently, these cells were incubated with secondary antibodies (Alexa Fluor 546-labeled anti-rabbit IgG) and the anti-mouse IgG (Alexa Fluor 488-labeled anti-mouse IgG) for 2 h and washed 3times by PBS. Finally, the cells were stained by DAPI for 15 min. Then, the cells imaging experiments were observed by fluorescence microscope.

### TUNEL Staining

Apoptotic cell death in the heart was detected *in situ* by terminal deoxynucleotidyl transferase (TdT)-mediated dUTP-biotin nick end-labeling (TUNEL) staining of fragmented DNA using an *In situ* Cell Apoptosis Detection Kit (Beyotime, Shanghai, China). The procedure was performed according to the manufacturer’s instructions. Briefly, cells were fixed with 4% paraformaldehyde and permeabilized by 0.3% Triton X-100 and then labeled by incubation (1 h, 37°C) with terminal deoxynucleotidyltransferase and nucleotide mixture containing fluorescein isothiocyanate-conjugated dUTP. The number of TUNEL positive cells was observed under the fluorescence microscope.

### Western Blot

The treated cells were collected and lysed with Radioimmunoprecipitation assay buffer (Beyotime, China). Then, the protein concentration was detected by the BCA protein assay kit (Pierce, IL, United States). Equal aliquots of protein (5 μg/ul, 10 μl) were heated at 100°C for 10 min and then fractionated by 10% SDS-PAGE gels. Then, they were put on the PVDF films and treated by TBS-T (with 1% BSA) for 2 h. The proteins were transferred electrophoretically from the gels to PVDF membranes, which were then treated with anti-rabbit CD36, PPARα, dynamin-related protein 1 (Drp1), Bax, Bcl-2 antibodies, and with anti-mouse GAPDH, antibodies overnight at 4°C. Subsequently, they were treated with a corresponding secondary antibody for 2 h. Immunoreactive proteins were revealed using an enhanced chemiluminescence kit (Pierce Chemical, Rockford, IL, United States). Expression of GAPDH was used as the control. The autoradiograms were carried out on an Alpha Innotech Photodocumentation System (Alpha Innotech, Hayward, CA, United States). The relative absorbance of the bands, which representing the amount of protein expression, was analyzed using Quantity One software (Bio-Rad Laboratories).

### Statistical Analysis

All experiments have performed a minimum of three times, and the data were analyzed using GraphPad Prism 8 software (GraphPad Software, Inc.). Data are presented as the means ± SD and were analyzed using a t-test. *p* < 0.05 was considered to indicate a statistically significant difference.

## Results

### Morphology and Properties of AuCur

**FIGURE 1:**
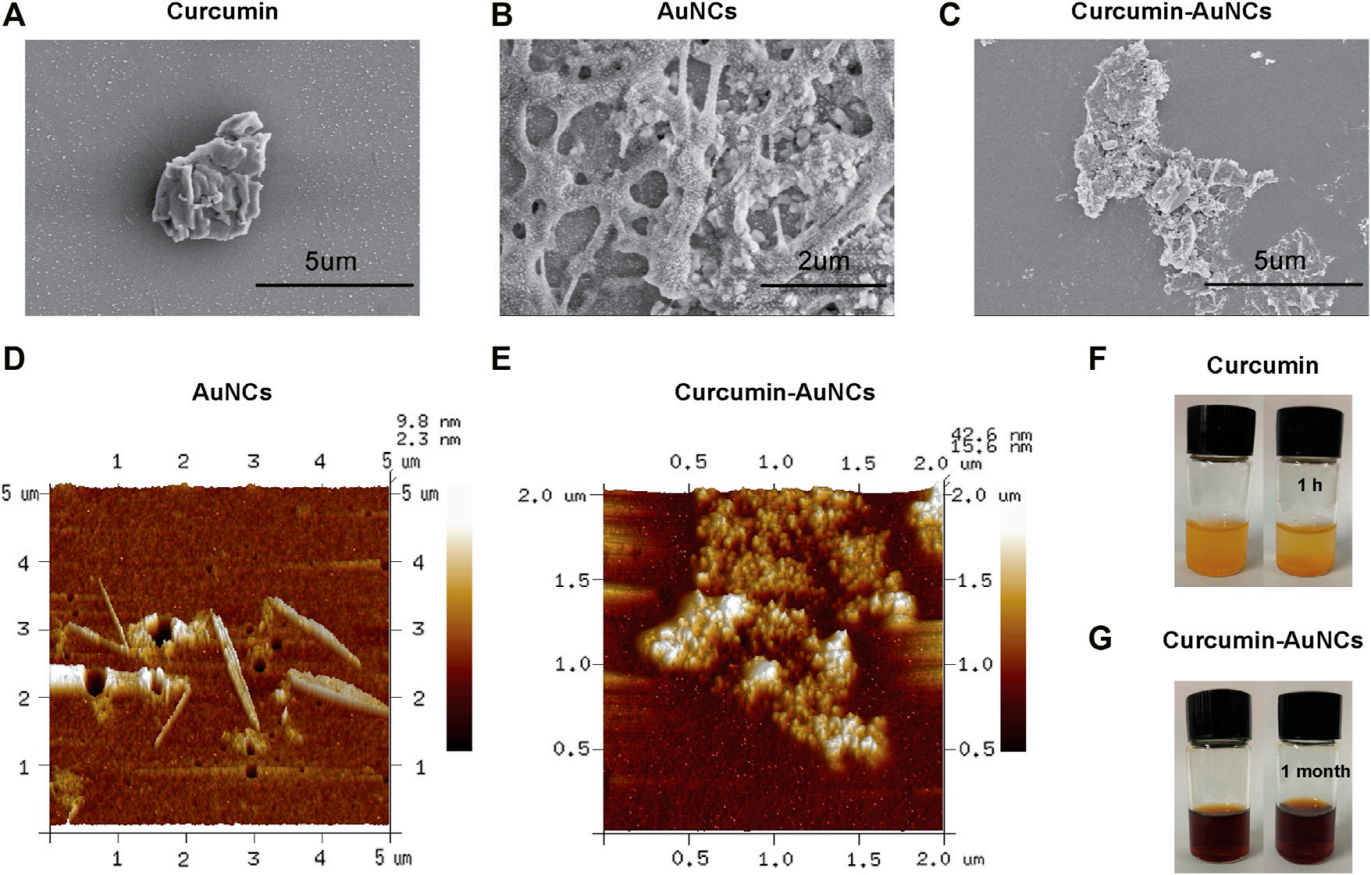
SEM of Cur **(A)**, AuNCs **(B)** and AuCur **(C)**. AFM of AuNCs **(D)** and AuCur **(E)**. Cur **(F)** and AuCur **(G)** in water from the beginning to 1 h. Au indicates AuNCs.

For comparison of Cur and AuCur, the synthesized product was characterized by Scanning Electron Microscope (SEM) and Atomic Force Microscope (AFM). SEM images showed that Cur (Figure 1A) exhibited as large aggregates, indicating Cur themselves were not well-dispersed. On the other hand, with the assistance of AuNCs (Figure 1B), AuCur is homogenously dispersed as small particles (Figure 1C), which will facilitate the adsorption efficiency of AuCur in the body. The high-magnification morphology of AuCur and AuNCs was further characterized by AFM. It presented that Cur was not uniformly distributed (Figure 1D), whereas uniformly dispersed small particles (Figure 1E) could be observed in the AuCur samples. We further found that Cur precipitated as large aggregates within 1 hour (Figure 1F), whereas AuCur remained stable after a month (Figure 1G). This reveals the poorly dispersed and unstable properties of Cur in contrast to AuCur, which is in agreement with SEM and AFM studies. Since the unstable drugs may not survive the bio culture, the more stable AuCur are more promising for long-term use.

### Toxicity Investigation

**FIGURE 2:**
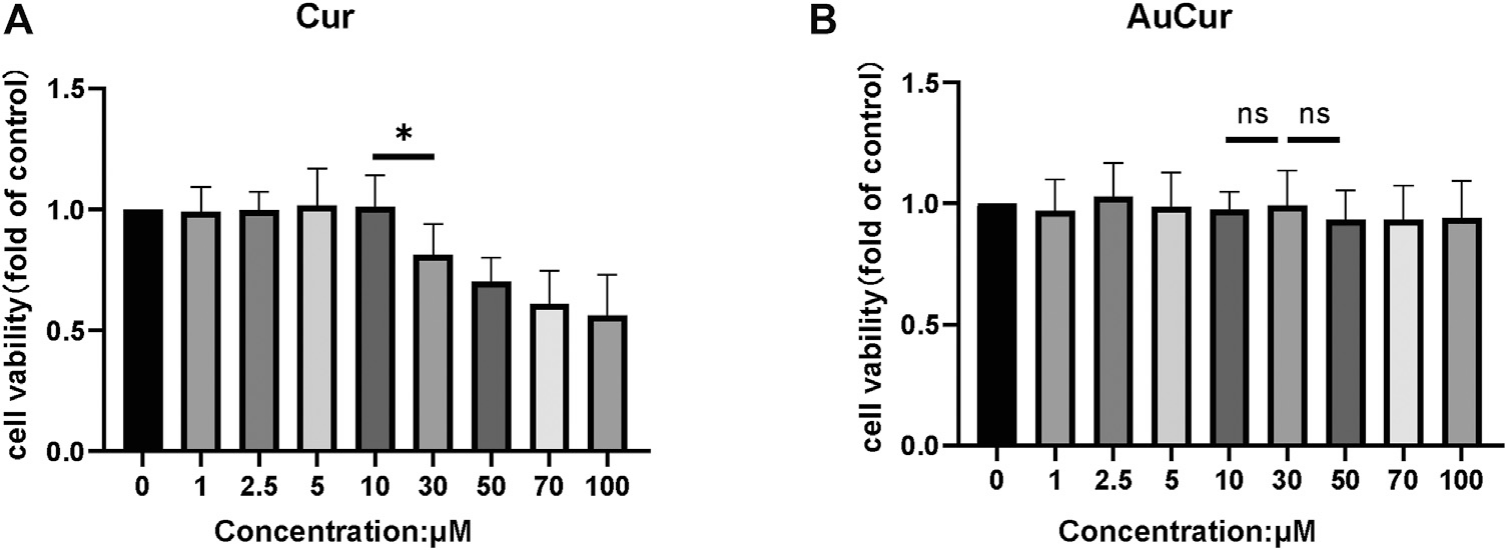
Cell viability analysis of Cur and AuCur. **(A)** The MTT of H9C2 cells for 24 h was treated with Cur. **(B)** MTT of H9C2 cells for 24 h was treated with AuCur. *p < 0.05. ns: Not Statistically Significant. Cur: curcumin, AuCur: Curcumin-AuNCs.

0–100 μM of the agents including the Cur group and the AuCur group was used for cell viability assay to check the drug toxicity. The cells used for the assay were myocardial cell line H9C2. After 24 h of drug (Cur) intervention, the cell viability decreased significantly at concentrations greater than 10 μM of Cur (Figure 2A). So, Cur with a concentration of 10 μM was selected for further experiments. In the case of AuCur, tested at same conditions than Cur, concentrations greater than 10 μM did not have a significant effect on cell viability. Indeed, AuCur remained safe up to 100 μM (Figure 2B).

### Effect of Lipid Accumulation

**FIGURE 3:**
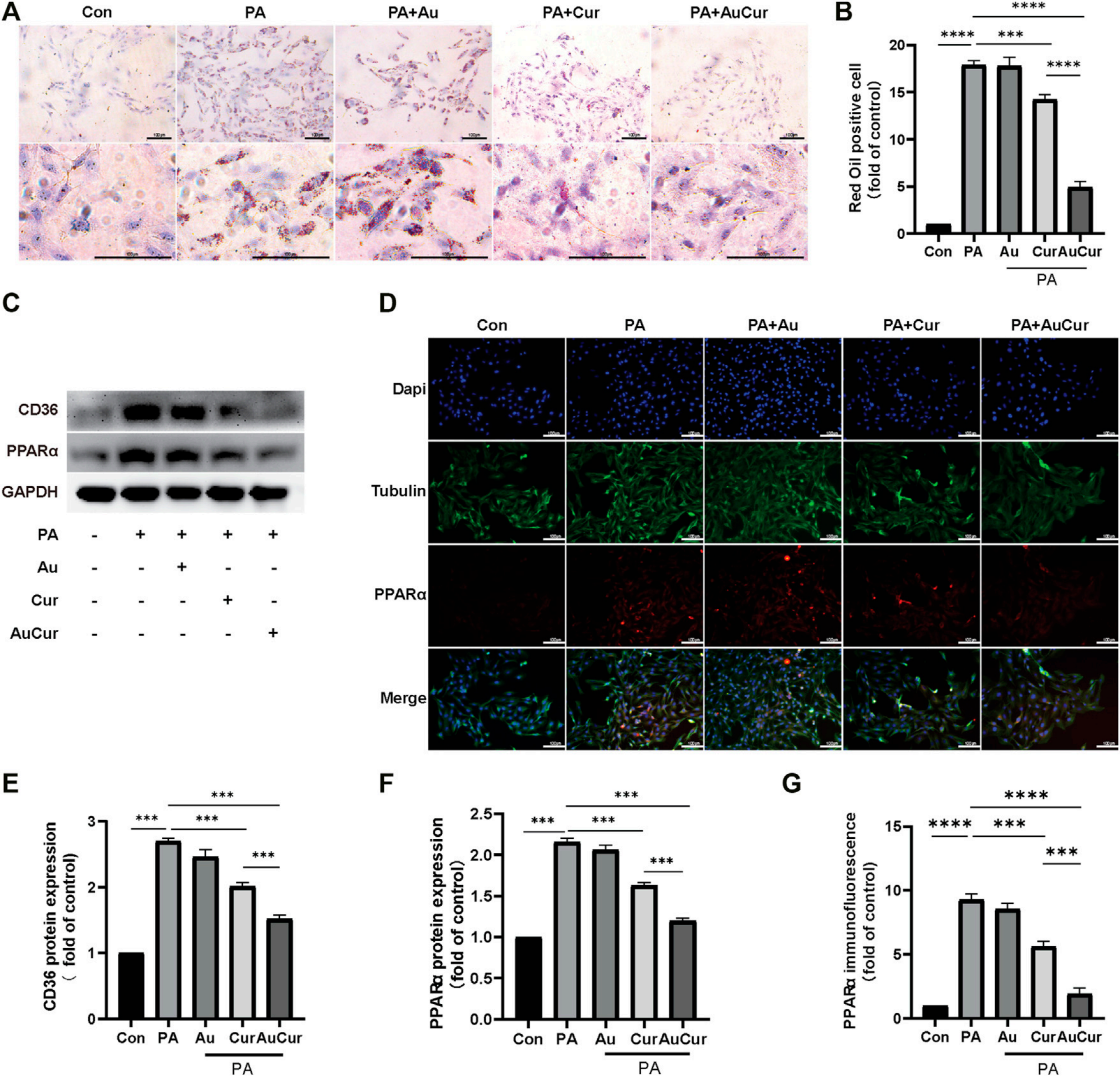
AuCur was more effective in reducing lipid accumulation than Cur alone. **(A)** Myocardial H9C2 cells were stained with oil red O treated with Control, PA, PA + Au, PA + Cur ,and PA + AuCur, respectively. Scale bar: 100 μm. **(C)** The expression of CD36 and PPARα was detected by WB. GAPDH was used as an internal reference. **(D)** PPARα fluorescence images of Control, PA, PA + Au, PA + Cur, and PA + AuCur groups. **(B)** Oil red O lipid droplet analysis. **(E–F)** WB band analysis. **(G)** PPARα fluorescence quantitative analysis. Scale bar: 100 μm *p < 0.05.

To study the reduction of lipid accumulation In H9c2 cells, H9c2 cells were pretreated with 0.3 μM palmitic acid (PA) for 24 h. Then they were divided into five groups: control group, PA group, Au group, Cur group, and AuCur group (The last three groups were all added to PA). The reducing of lipid accumulation rate of the Cur group and AuCur group was significantly higher than that of the palmitic acid group. In addition, AuCur had a better effect than Cur in reducing lipid accumulation, and the difference was statistically significant (Figures 3A,B). The result was shown in the WB experiment. After palmitic acid treatment, the gray value of the bands increased but decreased after drug treatment. And the result shows that the gray value of AuCur is lower than using Cur (Figures 3C,E,F). As can be seen from the immunofluorescence images, the fluorescence intensity of PA group increased, while that of the Cur group and AuCur group decreased, and the fluorescence intensity of AuCur was weaker than that of Cur group. The difference was statistically significant (Figures 3D,G). The expression of the Lipid transport factor PPARα has increased in PA cultured H9c2 cells. Both Cur and AuCur can ameliorate these phenomena. This indicates that Cur and AuCur block the overexpression of lipid transporters and have a therapeutic effect on lipid accumulation caused by high lipids Therefore, AuCur is more effective at preventing PPARα from overexpressing than Cur.

### Oxidative Stress and Mitochondrial Division

**FIGURE 4:**
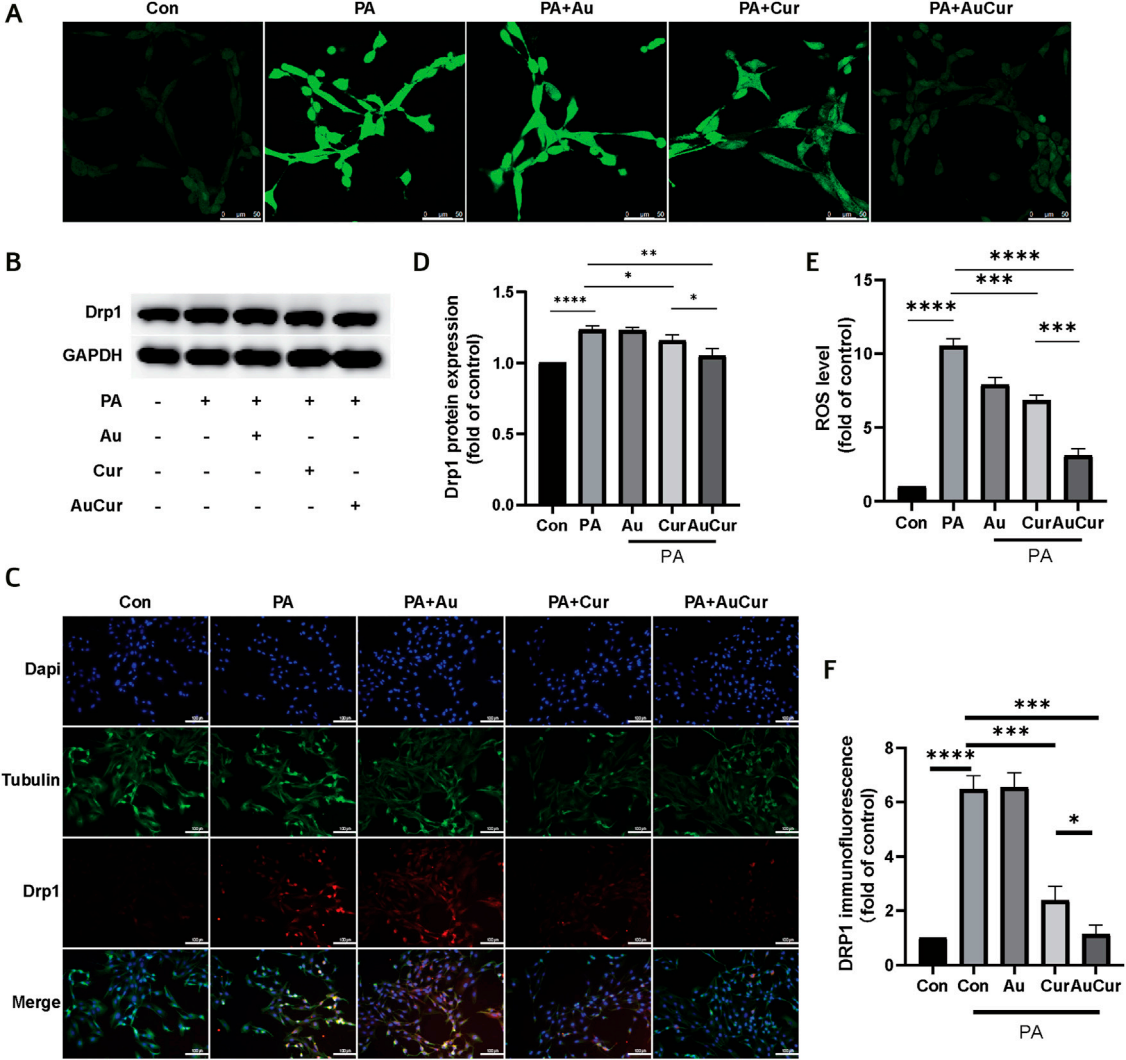
Effects of AuCur on ROS and mitochondria induced by PA action. **(A)** ROS confocal staining images of Control, PA, PA + Au, PA + Cur, and PA + AuCur. Scale bar: 50 μm. **(B)** Drp1 expression was detected by WB. GAPDH was used as an internal reference. **(C)** Drp1 fluorescence analysis of Control, PA, PA + Au, PA + Cur, and PA + AuCur groups. Scale bar: 100 μm. **(D)** Banding analysis of Drp1. **(E)** ROS image analysis. **(F)** Drp1 fluorescence image analysis. *p < 0.05.

To investigate the changes in H9C2 cells after palmitic acid treatment, we detected the changes in ROS levels. After adding 0.3 mM PA to cardiomyocytes for 24 h, ROS was detected with DCFH-DA. The results showed that the fluorescence intensity after palmitic acid treatment was significantly increased compared with that of the untreated group, indicating that the ROS level was significantly increased. The other three groups were treated with PA and then treated with three different drugs. The results showed that the fluorescence intensity of the Au group was no significant change. Both the Cur group and the AuCur group had therapeutic effects, and the treatment effect of the AuCur group was better than that of the Cur group, with statistical significance (Figures 4A,E). According to the above results, Cur slightly reduced the ROS, in contrast to AuCur which induce a significant reduction of the ROS. Then we explored changes in the level of mitochondrial fission-related factor Drp1. In the WB experiment, Drp1 increased after PA treatment, Cur and AuCur prevented this phenomenon, and AuCur showed a higher therapeutic effect (Figures 4B,D). Similarly, immunofluorescence results showed that Drp1 was significantly increased after PA treatment. After treatment with Au, Cur, and AuCur, the results showed that AuCur significantly reduced the fluorescence intensity, while the effect of Cur was not as effective as that of AuCur (Figures 4C,F). These results showed that Cur prevented the abnormal increase in the expression of the mitochondrial division factor Drp1 and was more effective when clumped with gold nanoparticles.

### Effects of AuCur on Apoptosis

**FIGURE 5:**
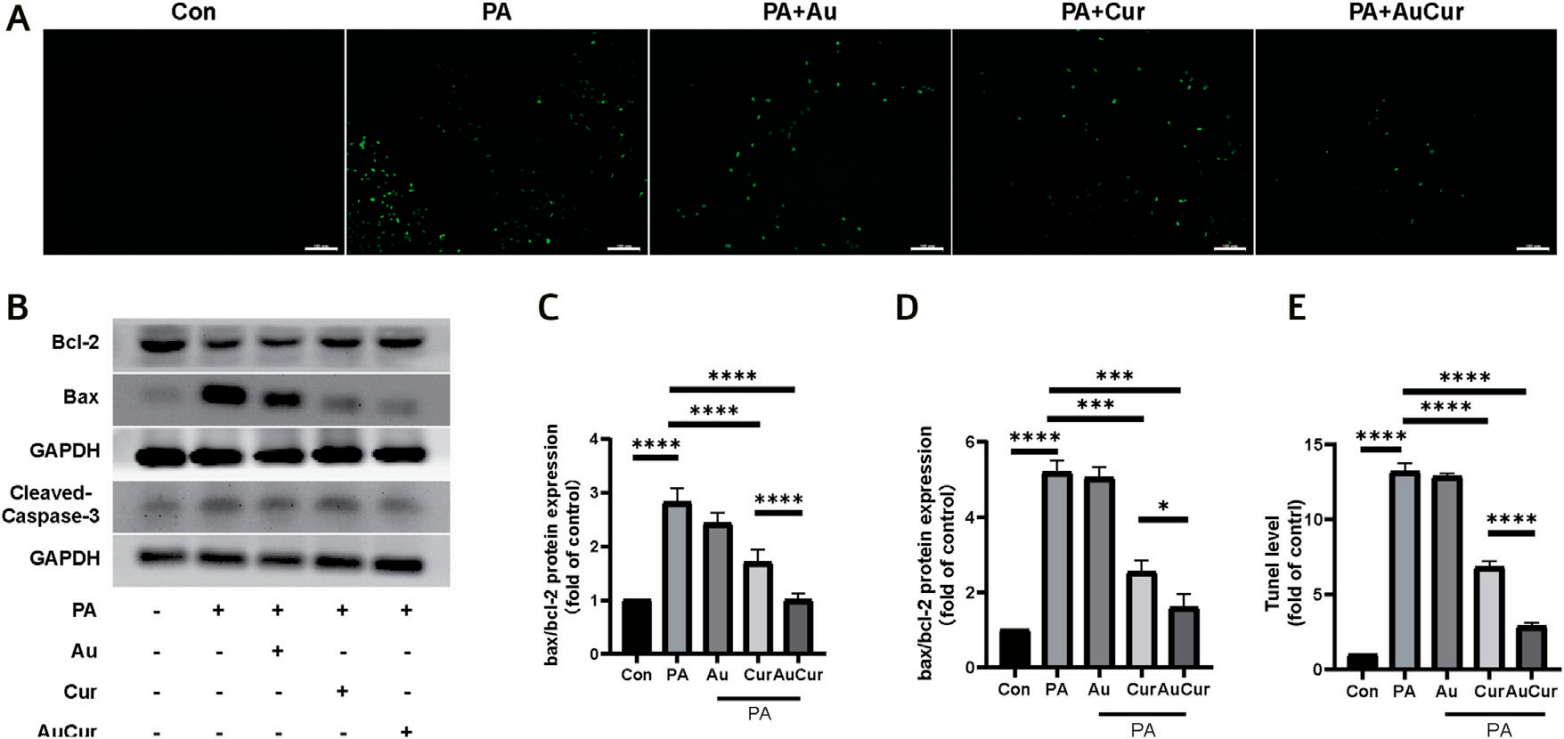
Effects of AuCur on apoptosis of myocardial cells induced by PA. **(A)** TUNEL images of Control, PA, PA + Au, PA + Cur, and PA + AuCur. Scale bar: 100 μm. **(B)** WB was used to detect the expression of Bax, Bcl-2, and Cleaved-caspase-3. GAPDH was used as internal reference. **(C–D)** Analysis of Bax, Bcl-2, Cleaved caspase-3 bands. **(E)** Image analysis by TUNEL. *p < 0.05.

In the fluorescence assay that could mark apoptotic cells, the number of apoptotic cells labeled green after treatment with palmitic acid increased, and the green fluorescence was decreased after treatment with Cur and AuCur, and AuCur showed better anti-apoptotic effect than Cur alone (Figures 5A,E). Then we used WB assay to detect three key proteins related to apoptosis and analyze their expression levels. Caspase-3 and Bax are closely related to apoptosis, and their expression is increased when apoptosis occurs, while Bcl-2 is on the contrary. We showed that the ratios of Caspase-3 and Bax to Bcl-2 increased after palmitic acid treatment compared with the untreated group, while both Cur and AuCur prevented this phenomenon, and AuCur showed better effect than Cur (Figures 5B–D). Therefore, AuCur was more effective than Cur in preventing the PA-induced apoptosis of cardiomyocytes.

### Treatment of Lipid Accumulation

**FIGURE 6:**
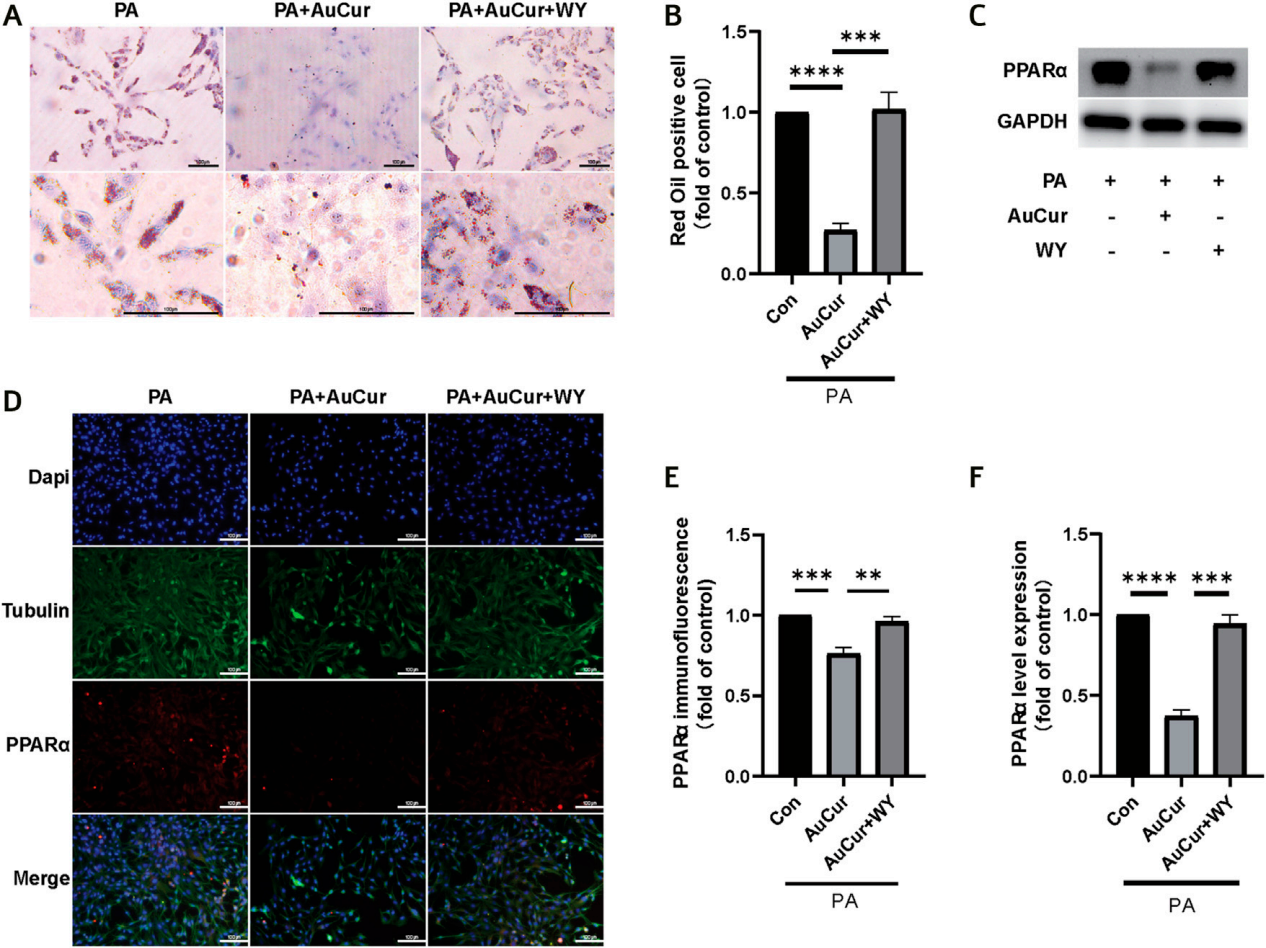
AuCur affects lipid accumulation by regulating PPARα. **(A)** Myocardial H9C2 cells were stained with oil red O treated with PA, PA + Cur, and PA + AuCur + WY, respectively. Scale bar: 100 μm. **(C)** The expression of PPARα was detected by WB. GAPDH was used as an internal reference. **(D)** PPARα fluorescence images of PA, PA + Cur, and PA + AuCur + WY groups. Scale bar: 100 μm. **(B)** Oil red fat droplet analysis. **(F)** PPARαWB banding analysis. **(E)** PPARα fluorescence image analysis. *p < 0.05.

We used PPARα agonist WY14643 to study the effect of AuCur on PPARα in the treatment of lipotoxic cardiomyopathy. We found that the number of intracellular lipid droplets decreased significantly after AuCur treatment, but increased with the addition of PPARα agonist WY14643 (WY) (Figures 6A,B). The expression of PPARα was detected by WB, and the experiment showed that the expression of PPARα was decreased after AuCur treatment, and the addition of WY14643 could counteract the therapeutic effect of AuCur (Figures 6C,F). The expression of PPARα was detected by immunofluorescence assay. As expected, the fluorescence intensity of PPARα decreased in the AuCur group, and the addition of WY14643 again neutralized the therapeutic effect (Figures 6D,E). PPARα, as a key factor in lipid metabolism, plays a key role in the treatment of DCM. The results showed that AuCur affected lipid accumulation by altering PPARα.

### Treatment of Myocardial Damage

**FIGURE 7:**
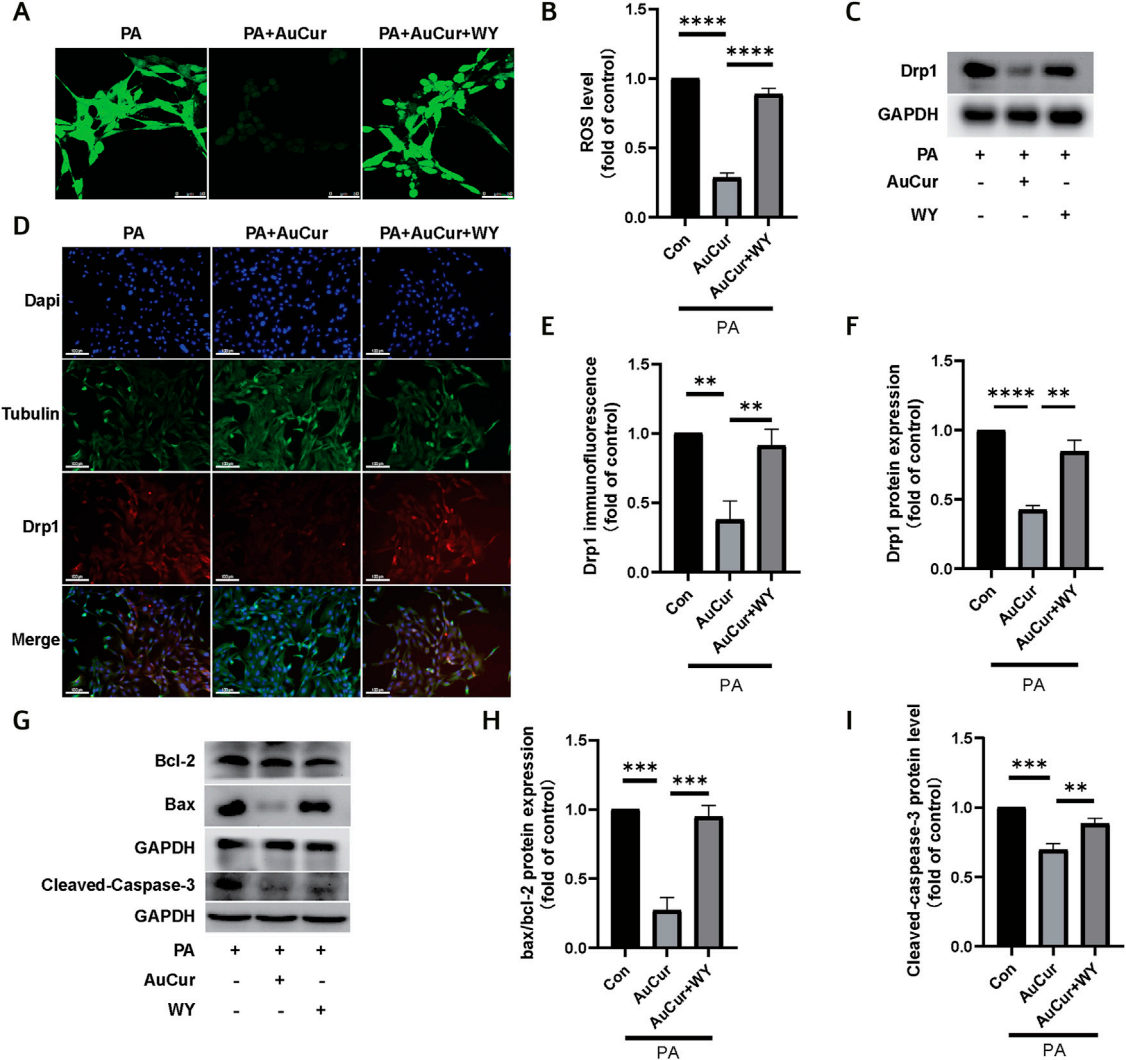
AuCur regulates PPARα to treat PA-induced ROS elevation, mitochondrial damage, and apoptosis. **(A)** ROS confocal staining images of PA, PA + AuCur, and PA + AuCur + WY. Scale bar: 50 μm. **(C)** Drp1 expression was detected by WB. GAPDH was used as an internal reference. **(D)** Drp1 fluorescence analysis of PA, PA + AuCur, and PA + AuCur + WY groups. Scale bar: 100 μm. **(B)** ROS image analysis. **(F)** Banding analysis of Drp1. **(E)** Drp1 fluorescence image analysis. **(G)** WB was used to detect the expression of Bax, Bcl-2, and Cleaved-caspase-3. GAPDH was used as an internal reference. **(H–I)** Bax, Bcl-2, and Cleaved-caspase-3 bands were analyzed. *p < 0.05.

To explore the role of PPARα in the treatment of high lipid-induced ROS elevation by AuCur, ROS fluorescence was filmed. The results showed that PA increased the green fluorescence of ROS, decreased the green fluorescence after AuCur treatment, and increased the green fluorescence after WY and AuCur combined treatment (Figures 7A,B). Similarly, in the immunofluorescence experiment of Drp1, PA increased Drp1 (red fluorescence), while the red fluorescence decreased after AuCur treatment, while the addition of WY increased the red fluorescence (Figures 7D,E). In the WB, Drp1 expression was enhanced under the action of PA, and AuCur inhibited this enhancement, but the inhibitory effect of AuCur was canceled under the action of WY (Figures 7C,F). In terms of the inhibitory effect of AuCur on apoptosis, we also found that AuCur combined with PPARα agonist had no therapeutic effect on the increase of Bax, a decrease of Bcl-2, and an increase of Caspase-3 induced by PA therapy (Figures 7G–I). After high-fat induction, ROS increased, while AuCur inhibited ROS, Drp1, and apoptosis. WY and AuCur simultaneously increased ROS, Drp1, and apoptosis again. These results suggested that the inhibitory effect of AuCur on palmitic acid-induced myocardial damage was neutralized by the PPARα agonist.

## Discussion

A high-fat diet can bring a variety of harms, such as inflammation, oxidative stress, and chronic diseases, etc. (Yang et al., 2021; Zeng et al., 2021) High blood lipids are closely related to cardiovascular diseases, which can lead to myocardial infarction, atherosclerosis, and other serious diseases (Li et al., 2018; Wang et al., 2021). The heart is an important place for the uptake and utilization of fatty acids, and the study of the effect of high lipid on the heart is of great importance. To ensure normal heart activities, a large amount of intake of fatty acids with low productivity and efficiency will lead to excessive accumulation of lipids and intracellular toxicity (Shiomi et al., 2013). The excessive accumulation of lipids can directly lead to the imbalance between the production and clearance of reactive oxygen species, which is the occurrence of oxidative stress reaction. Studies have shown that reducing ROS production protects cardiomyocytes damaged by glycolipid toxicity (Kolleritsch et al., 2020; Yang et al., 2020). In our study, AuCur successfully reduced the accumulation of lipid droplets and the increase of ROS in cardiomyocytes due to high lipid levels. Mitochondrial dysfunction can occur in lipotoxic cardiomyopathy. Mitochondria are remodeled by dividing and merging themselves (Vendrov et al., 2015). When Drp1 expression is increased, it means that mitochondrial division is increased, mitochondrial diameter becomes smaller, and mitochondrial function is impaired (Ni et al., 2015; Tsushima et al., 2018; Xue et al., 2019). In the experiments under high lipid-induced the expression of Drp1 did increase, but AuCur stopped Drp1 induced by high-fat rise, indicating the effective inhibition of mitochondrial division factor in the treatment of lipotoxic cardiomyopathy, which further protects mitochondria in the treatment of lipotoxic cardiomyopathy. Lipid accumulation, oxidative stress, mitochondrial damage, and other factors involved in the development of cardiomyopathy can promote the occurrence of myocardial cell apoptosis (Mishra and Chan, 2016). When glucose and lipid metabolism is abnormal, the rate of apoptosis of cardiomyocytes becomes very high (Huynh et al., 2013). Apoptosis of cardiomyocytes in cardiomyopathy was the worst result, but our drug successfully reduced the cell apoptosis, indicating that our drug successfully played a role in the treatment of cardiomyopathy.

AuCur retains Cur’s pharmacological efficacy and enhances its biocompatibility, making it more readily absorbed and utilized. And because Cur can treat a variety of chronic diseases (Ho et al., 2000; Panahi et al., 2015; Kunnumakkara et al., 2017; Parsamanesh et al., 2018). Next, we can continue to study the effect of AuCur on other diseases.

In a high lipid environment, lipid transport and utilization increased (Wright et al., 2009; Maiti and Dunbar, 2018). As a lipid transporter, CD36 increases the burden of cardiac lipid metabolism when it is overexpressed (Dirkx et al., 2014). AuCur was able to reduce the abnormal increase of CD36 in our experiment. PPARα is a key factor in lipid transport and uptake. Cardiac-specific expression of PPARα in animal models can induce abnormal lipid metabolism, mimicking the phenotype of lipotoxic cardiomyopathy. The knockout of PPARα effectively prevented the cardiotoxicity caused by lipid metabolism (Glatz and Luiken, 2018; Wu et al., 2018). Therefore, the regulation of PPARα is the key to the treatment of lipotoxic cardiomyopathy. Increased ROS and mitochondrial splitting apoptosis brought about by lipotoxicity accelerate the progression of DCM Mitochondrial fragmentation and dysfunctional mitochondria are produced after excessive mitosis, which is related to ROS regulation of increased Drp1 expression (Chen et al., 2021; Feng et al., 2019). Although Cur can alter this disease state, its biocompatibility limits the efficacy, and AuCur outperforms Cur in treating the disease. Our previous experiments showed that AuCur inhibited the abnormal elevation of PPARα in hyperlipidemia and ameliorated other deleterious effects of lipid toxicity on the myocardium. In our experiment, WY14643 was added to co-act with AuCur, and it was found that WY14643 could counteract the therapeutic effect of AuCur on the abnormal accumulation of lipid droplets in cardiomyocytes and other damages. These results indicated that this drug does regulate PPARα, and further realizes the treatment of lipotoxic cardiomyopathy by regulating PPARα.

## Conclusion

AuCur have improved the biocompatibility and the efficacy of Cur and are promising to relieve the symptoms of high fat-induced cardiomyocytes. The therapeutic effect is mainly related to the enhanced efficiency for the regulation of lipid accumulation, PPARα, and ROS. The application of AuNCs for improving drug efficiency provides a new venue to treat diabetic cardiomyopathy.

## Data Availability

The original contributions presented in the study are included in the article/Supplementary Material, further inquiries can be directed to the corresponding author.
